# Amantadine for NeuroenhaNcement in acutE patients Study - a protocol for a prospective pilot proof of concept phase IIb study in intensive and intermediate care unit patients (*ANNES*)

**DOI:** 10.1186/s12883-023-03345-w

**Published:** 2023-08-22

**Authors:** Anna Hofmann, Corinna Blum, Constanze Single, Kamal Adeyemi, Patricia Schwarz, Vasileios Siokas, Tim W. Rattay, Helene A. Häberle, Reimer Riessen, Bettina Brendel, Iris Haug, Ruth Bösel, Manola Zago, Peter Martus, Ulf Ziemann, Annerose Mengel, Katharina Feil

**Affiliations:** 1 Department of Neurology/neurodegenerative diseases, University Hospital Tübingen, Tübingen, Germany; 2 Department of Neurology & Stroke, University Hospital Tübingen, Tübingen, Germany; 3 Department of Neurology, Faculty of Medicine, School of Health Sciences, University Hospital of Larissa, University of Thessaly, Larissa, 41100 Greece; 4 Department of Anesthesiology and Intensive Care Medicine, University Hospital Tübingen, Tübingen, Germany; 5 Department of Medicine, Medical Intensive Care Unit, University Hospital Tübingen, Tübingen, Germany; 6 Institute for Clinical Epidemiology and Applied Biometry, Faculty of Medicine, Eberhard-Karls- University Tübingen, Tübingen, Germany; 7 Center for Clinical Studies ZKS Tübingen, University Hospital Tübingen, Tübingen, Germany; 8 Hertie Institute for Clinical Brain Research, University of Tübingen, Tübingen, Germany

**Keywords:** Amantadine, Reduced consciousness, Coma, Neurointensive care, Stroke

## Abstract

**Background:**

Persisting coma is a common complication in (neuro)intensive care in neurological disease such as acute ischemic stroke, intracerebral hemorrhage or subarachnoid hemorrhage. Amantadine acts as a nicotinic receptor antagonist, dopamine receptor agonist and non-competitive N-Methyl-D-aspartate receptor antagonist. Amantadine is a long-known drug, originally approved for treatment of influenza A and Parkinson`s Disease. It has been proven effective in improving vigilance after traumatic brain injury. The underlying mechanisms remain largely unknown, albeit anti-glutamatergic and dopaminergic effects might be most relevant. With limited evidence of amantadine efficacy in non-traumatic pathologies, the aim of our study is to assess the effects of amantadine for neuroenhancement in non-traumatic neurointensive patients with persisting coma.

**Methods:**

An investigator-initiated, monocenter, phase IIb proof of concept open-label pilot study will be carried out. Based on the Simon design, 43 adult (neuro)intensive care patients who meet the clinical criteria of persisting coma not otherwise explained and < 8 points on the Glasgow Coma Scale (GCS) will be recruited. Amantadine will be administered intravenously for five days at a dosage of 100 mg bid. The primary endpoint is an improvement of at least 3 points on the GCS. If participants present as non-responders (increase < 3 points or decrease on the GCS) within the first 48 h, the dosage will be doubled from day three to five. Secondary objectives aim to demonstrate that amantadine improves vigilance via alternative scales. Furthermore, the incidence of adverse events will be investigated and electroencephalography (EEG) will be recorded at baseline and end of treatment.

**Discussion:**

The results of our study will help to systematically assess the clinical utility of amantadine for treatment of persisting coma in non-traumatic brain injury. We expect that, in the face of only moderate treatment risk, a relevant number of patients will benefit from amantadine medication by improved vigilance (GCS increase of at least 3 points) finally leading to a better rehabilitation potential and improved functional neurological outcome. Further, the EEG data will allow evaluation of brain network states in relation to vigilance and potentially outcome prediction in this study cohort.

**Trial Registration:**

NCT05479032.

**Supplementary Information:**

The online version contains supplementary material available at 10.1186/s12883-023-03345-w.

## Introduction

### Background and rationale

Disorders of consciousness (DoCs) can be categorized into coma, vegetative state (VS)/unresponsive wakefulness syndrome (UWS) and minimally conscious state (MCS). Coma is a state of unawareness from which the patient cannot be aroused [1]. The classical definition includes absent of sleep-wake cycles and closed eyes [1]. The vegetative state or unresponsive wakefulness syndrome (UWS) is denoted as wakefulness without awareness. These patients are eyes-opened with only reflexive movements and do not obey commands [2]. Patients in MCS show cognitively mediated, non-reflexive behaviors, which are related to relevant environmental stimuli. The occurrence of this behavior is inconsistently, but reproducible [3]. However, some comatose patients show eye-opening early after brain injury, but do not meet the criteria for UWS. These patients are newly classified as patients with “eye-open coma” [4].

Disorders of consciousness are a quite common complication in (neuro)intensive care, occurring in up to 60% of patients after acute ischemic stroke (AIS), intracerebral hemorrhage (ICH) and subarachnoid hemorrhage (SAH) [5–7]. Disorders of consciousness are associated with an increased risk of stroke-related complications (90% versus 67%) and a strong predictor for an unfavorable neurologic outcome as well as mortality [5, 8, 9]. Thus, disorders of consciousness undermine rehabilitation success and may also lead to unjustified intensive care therapy limitation [10].

On the neuroanatomical level, arousal is mediated by neuronal populations in the ascending reticular activating system and associated network, comprising of the rostral brain stem, tegmentum, diencephalon and projections to the cerebral cortex and the basal ganglia [11, 12]. Hence, all cerebral pathologies compromising these circuitries may lead to unconsciousness, although prognoses may differ [13]. Neurotransmitter modulation of human consciousness may be disrupted during acute brain injury [14] and alterations in glutamate, dopamine, acetylcholine, gamma aminobutyric acid (GABA) and orexin have been described providing a possible pharmacologic target to promote repair and restoration of neural pathways in disorders of consciousness [15]. To summarize, complex interactions of various neurotransmitter systems and brain regions are regulating alertness. Equivalently, amantadine is an agent with multiple mechanisms of action. Mainly, amantadine acts as a weak uncompetitive antagonist of the N-methyl-D-aspartate (NMDA) receptor, thereby decreasing the excitatory input, and may have direct and indirect effects on dopamine neurons [16, 17]. The increase in dopaminergic tone may additionally occur through a few potential mechanisms, including inhibition of dopamine reuptake; an increase in dopamine release from dopaminergic nerve endings; an increase of dopa decarboxylase activity; an increase of dopamine transporter activity; inhibition of NMDA-evoked release of acetylcholine; and an increase of dopamine D2 receptor availability [18, 19]. Amantadine functions furthermore on the glutamatergic pathways decreasing this excitatory input. All in all, the mechanism of action of amantadine has to be interpreted and evaluated in relation to its concentrations reached at a given target in humans following therapeutic doses and its affinity at the target [16]. The 2018 practice guidelines for disorders of consciousness provide a Level B recommendation for using amantadine in adults with traumatic disorders of consciousness at 4 to 16 weeks postinjury to promote functional recovery [20, 21]. Further, amantadine is used off-label in the clinical routine for treating persisting coma in non-traumatic (neuro)intensive care unit patients despite the lack of high-quality data supporting the efficacy of pharmacologic interventions targeting neurotransmitter pathways attempting to improve long-term functional outcome in these patients [22, 23]. However, the first report of amantadine administration in post-stroke patients suggested an improvement in intellectual, motor and emotional function [24]. Further, there exists extensive evidence from animal studies for the neuroprotective effect of anti-glutamatergic substances after hypoxic brain damage [25]. In more detail, it has been shown that dopamine activity is disturbed following acute stroke and that substitution may be beneficial [26].

### Trial rationale

The American Stroke Association recently determined that the benefit of neuro-stimulants for stroke-related cognitive impairment including disturbed vigilance is still unclear [27]. Consequently, a great and unmet need exists for controlled, prospective studies systematically investigating the effects of amantadine treatment in non-traumatic (neuro)intensive care patients with persisting coma. From a mechanistic point of view, even in this field a clinical benefit appears rather reasonable, which probably might help to improve the patients’ participation in early rehabilitation procedures and, therefore, the long-term neurological outcome. As the level of evidence is quite limited, especially within the context of non-traumatic pathologies, the aim of *Amantadine for NeuroenhaNcement in acutE patients Study* (ANNES) is to assess the potential use of amantadine for neuroenhancement in non-traumatic neurointensive care patients with persisting coma.

## Objectives

### Research hypothesis

Intravenous amantadine treatment over five days improves consciousness, defined as an increase in Glasgow Coma Scale (GCS) score of at least 3 points, in non-traumatic (neuro)intensive care unit patients.

### Study objectives and outcomes

#### Primary objective and endpoint

The primary endpoint is defined as an increase in GCS score of at least 3 points on visit 6 compared to visit 1 (screening visit).

The primary endpoint is assessed by two independent clinical investigators who separately score the patient. In case of disagreement, a third independent investigator will perform GCS score.

### Secondary objectives and endpoints

Secondary objectives are improvement of vigilance/awareness measured via alternative scales using the following: Richmond Agitation-Sedation Scale (RASS), Full Outline of UnResponsiveness (FOUR) Score Coma Scale, Glasgow Outcome Scale – Extended (GOS-E) and Coma Recovery Scale revised (CRSR). Further secondary outcome variables include functional outcome using National Institute of Health Stroke Scale (NIHSS) regarding potential stroke severity and modified ranking score (mRS) in follow-up after discontinuation of medication and after three months. The incidence of complications regarding delirium will be assessed using the Intensive Care Delirium Screening Checklist (ICDSC). The Montreal Cognitive Assessment (MoCA) will assess the neuropsychological outcome. Regarding validity of these measures for determining outcome, please see the paragraph below.

Any adverse event (AE) (any untoward medical occurrence including an abnormal laboratory finding, regardless of its causal relation to the study treatment) will be recorded during the whole study period.

### Clinical assessment scales

#### Consciousness

Different rating scales will quantify the clinical severity of disorders of consciousness.

The GCS rates patients based on their ability to perform limb and eye movements as well as to speak. These three categories represent the core elements of the scale: „eye“, „verbal“, and „motor“. A person’s GCS score can range from 3 (completely unresponsive) to 15 (completely responsive). This score is fast, easily and reliably to perform and can be used in emergency situations and also to monitor hospitalized patients. Regarding the assessment of amantadine treatment effects, it has already been used in the context of traumatic brain injury [28] as well as within a retrospective analysis of Leclerc et al. (2021) in post-stroke patients [29].

The FOUR Score is a 17-point scale with possible scores ranging from 0 to 16 (decreasing score is associated with a worsening level of consciousness). The FOUR score overcomes some of the shortcomings of the GCS by assessing the four domains of neurological function: eye responses, motor responses, brainstem reflexes, and even breathing pattern [30].

The RASS is a ten-step scale for assessment and quantification of sedation as well as agitation with the value ‘0’ describing the physiological state [31].

The GOS-E is a scale for patients with brain injuries, such as cerebral traumas or strokes that groups the victims by the objective degree of recovery [32]. Later, the same authors proposed to split the 3 better categories (severe disability to good recovery, i.e., 3 to 5) in lower and upper sub-categories, leading to the extended version of the scale (GOS-E) which includes 6 plus 2 (death and vegetative state), i.e., 8 categories in total [33].

The CRS-R is a standardized neurobehavioral assessment instrument for the usage in patients with disorders of consciousness. It is intended to establish diagnosis, monitor recovery, predict outcome as well as assess treatment effectiveness [34].

#### Delirium

A clinician can easily and quickly apply the ICDSC. The ICDSC can easily and quickly be applied by a clinician or a nurse in the critical care setting to screen all patients for a delirium (even when communication is compromised in case of aphasia) [35].

### Stroke severity and outcome

The mRS is a commonly used scale for measuring the degree of disability or dependence in the daily activities of people who have suffered a stroke or other causes of neurological disability [36].

The NIHSS is used to objectively quantify the clinical impairment caused by a stroke. The NIHSS is composed of 11 items, each of which scores a specific ability between 0 and 4. For each item, a score of 0 indicates normal function, while a higher score is indicative of some level of impairment. Each item’s individual scores are summed to calculate a patient’s total NIHSS score. The maximum possible score is 42 [37].

#### Neurocognition

The MoCA is a widely used screening assessment for detecting cognitive impairment [38]. It was validated in the setting of mild cognitive impairment, and has subsequently been adopted in numerous other clinical settings.

### Questionnaire for therapists

The Early Functional Abilities (EFA) Scale is a valid instrument for nursing staff and physiotherapists to evaluate subtle clinical changes in early neurological rehabilitation. It includes 20 items in 4 categories (autonomic, oro-facial, sensorimotor and cognitive functions/abilities). Each item is rated on a five-point-scale: “1-no function”, “2-severe disturbance”, “3-moderate disturbance”, “4-slight disturbance” and “5-normal”. EFA total score ranges from 20 (worst) to 100 (best). In early rehabilitation stage, independence in activities of daily life (ADL) is not an appropriate measurement for therapeutic progress (e.g., Barthel-Index). Other frequently used assessments for early rehabilitation (e.g., GCS) focus on wakefulness, more specifically reactions to sensory stimuli. The EFA scale combines both, activities of daily life (ADL) such as wakefulness (cognitive functions) and therefore allows a differentiated assessment of the patient abilities [39].

### Neurophysiological assessment including EEG

A resting-state electroencephalography (rsEEG) with eyes closed and (if possible) open for three minutes each will be performed using a 64-channel gel filled sintered electrode EEG cap (EasyCap, Munich, Germany) and an optically isolated battery-powered biosignal amplifier (Brain Products, Gilching, Germany). The electrode impedances will be maintained below 5 kΩ during recording. A hardware lowpass filter will be set to 1 kHz with a sampling rate of 5 kHz.

The EEG data will be analyzed by calculating the power spectrum for fast (alpha (8–13 Hz), beta (14–30 Hz)) and slow oscillations (theta (4–7 Hz), delta (1–3 Hz)) as well as functional connectivity and oscillatory microstates, since faster oscillations (especially alpha power), better functional connectivity and more diversity and variability in the brain activity were predictive for emerging from disorders of consciousness [40, 41]. Specifically, better thalamocortical and frontoparietal connectivity was associated with less impaired consciousness.(Bai et al., 2021; Edlow et al., 2021; King et al., 2013) Connectivity will be assessed using, for instance, coherence. Microstates are EEG patterns that persist for a few seconds and correspond to thought processes. They are thus a direct expression of consciousness.(Lehmann, 1990) Furthermore, EEG data will be manually inspected for epileptic activity.

EEG is performed three times during the study. First, at baseline visit (before amantadine treatment) in order to detect epileptic EEG-activity, that exclude the patient from the study. The further EEGs are performed on Visit 6 (after 120 h on amantadine treatment) and Visit 7 (14 days after the last amantadine dose).

### Laboratory examinations

Laboratory examinations are carried out at the central laboratory of the University Hospital Tübingen. A blood sample will be taken from each patient to exclude liver or kidney failure, and a pregnancy test for women of child-bearing potential will be performed. A pregnancy test is not required for postmenopausal (amenorrhea > 12 months), surgically sterilized or hysterectomized women. The following laboratory parameters will be measured (but not routinely documented in the CRF): sodium, potassium, creatinine, serum bilirubin level, AST, ALT, urea, ALP, TSH, hemoglobin, erythrocytes, hematocrit, thrombocytes and leukocytes. In the case of an AE, laboratory parameters will be documented. The total amount of blood taken per subject during the entire trial will be approximately 10 ml.

### Training plans

The study personnel will be trained in the entire course of the study, study requirements as well as standardized usage of questionnaires (e.g., CRS-R, FOUR Coma Scale). The training will be monitored in a training log.

### Trial design

The *Amantadine for NeuroenhaNcement in acutE patients Study* (ANNES) is designed as an investigator-initiated, monocenter, phase IIb, proof of concept, open-label pilot study. It is a single-group trial assessing the improvement of consciousness of 43 adult (neuro)intensive patients after five days of Amantadine therapy (dosage of 100 mg twice daily). The timing between brain injury, disorder of consciousness and administration of amantadine is not fixed in ANNES. However, the treatment initiation is limited to intensive and intermediate care units and all other reasons for reduced consciousness have to be excluded (e.g., status epilepticus, sepsis, electrolyte imbalance, hyperglycemia, akinetic crisis and delirium). The study was initiated at the Department for Neurology at the University Hospital Tübingen. The study has been approved by the local ethics committee (protocol no. 2021-10). Furthermore, the study has been approved by the legal medical regulatory authorities (Federal Institute for Drugs and Medical Devices in Germany: *Bundesinstitut für Arzneimittelsicherheit und Medizinprodukte*, BfArM in Germany). ANNES is conducted in accordance with the International Conference for Harmonization of Technical Requirements for Pharmaceuticals for Human Use – Good Clinical Practice Guideline (ICH-GCP) and the Declaration of Helsinki. The trial has been prospectively registered at www.clinicaltrialsregister.eu (EudraCT no. 2022-002418-18) and https://www.clinicaltrials.gov (NCT05479032). Patient insurance for the study has been arranged (policy number 5,701,031,103,013).

## Methods: participants, interventions and outcomes

### Study setting

Recruitment will take place on the intensive and intermediate care units within the department of neurology, internal medicine and anesthesiology of the University Hospital Tübingen (UKT) in Germany.

### Patient population and eligibility criteria

Patients are screened for eligibility according to the inclusion and exclusion criteria (for detailed description, see Table 1). All relevant medical and non-medical conditions should be considered when deciding whether the study is suitable for a particular patient. A screening sheet is used to assess the persisting, not otherwise explained coma to ensure that relevant differential diagnoses have been excluded. To be eligible, patients must be aged 18 years or older with prolonged (≥ 72 h) and persisting coma not otherwise explained with a Glasgow Coma Scale (GCS) < 8. The exclusion criteria for this study were chosen according to the official contraindications for amantadine [42]. In addition, patients with unresponsive wakefulness (UWS) or minimally conscious state (MCS) are excluded. As the investigated study collective is per definition suffering from reduced consciousness, the patient’s legal representative will provide written informed consent. No gender ratio has been stipulated in this trial as one previous clinical study did not indicate any gender difference in the effect of the trial treatment in terms of efficacy and safety [43].

**Table 1:** Inclusion and exclusion criteria

Inclusion criteria	Details
Coma with reduced consciousness with GCS < 8	Lasting at least 72 h, not otherwise explained
Age ≥ 18 years	at the time of signing the informed consent.
Inconspicuous EEG and ECG	-
In woman of child bearing potential: pregnancy excluded	By measurement of human chorionic gonadotropin (hCG) in serum before start of study medication
Informed consent	As subjects per definition suffer from reduced consciousness and therefore are not in a position to provide informed consent themselves, prior to any study related procedures, the patient’s legal representative has to give written informed consent. The informed consent of the patient will be sought retroactively as soon as possible.
Exclusion criteria	
Reduced consciousness, otherwise sufficiently explained	Such as reduced consciousness due to status epilepticus, hyperglycemia, electrolyte imbalance, hyperkaliemia, akinetic crisis in Parkinson’s Disease or Delirium (Intensive Care Delirium Screening Checklist (ICDSC) > 4 or > 5 in aphasic patients)
Age < 18 years	**-**
Participation in other interventional study	Participation in an observational trial is acceptable
History of epileptic seizures/status epilepticus	-
Women during pregnancy and lactation	-
History of hypersensitivity to the investigational medicinal product	Or to any drug with similar chemical structure or to any excipient present in the pharmaceutical form of the investigational medicinal product
Concomitant therapy with memantine	-
Severe uncompensated heart failure (NYHA IV)	E.g., due to cardiomyopathy or myocarditis
Atrioventricular block (AV block) second-degree and third-degree	-
Known bradycardia (below 55 beats/minute)	-
Known long QT interval (QTc according to Bazett > 420 ms) or recognizable U-waves or congenital QT syndrome in the Family history	-
History of serious ventricular arrhythmias, including torsade de pointes	-
Hypokalemia or hypomagnesemia	-
Concomitant therapy with Budipine or other QT-prolonging drugs	Class IA (quinidine, disopyramide, procainamide) and III (amiodarone, sotalol) antiarrhythmic drugs, antipsychotics (thioridazine, chlorpromazine, haloperidol, pimozide), tri- and tetracyclic antidepressants (amitriptyline), antihistamines (astemizole, terfenadine), macrolide antibiotics (erythromycin, clarithromycin), gyrase inhibitors (sparfloxacin), azole antifungals and other medicines such as halofantrine, cotrimoxazole, pentamidine, cisapride, bepridil
Impaired renal function, measured by glomerular filtration rate (GFR) < 10 ml/min	Impaired renal function will necessitate dose adjustment (see Table 3).

### Study procedures

**Fig. 1:**
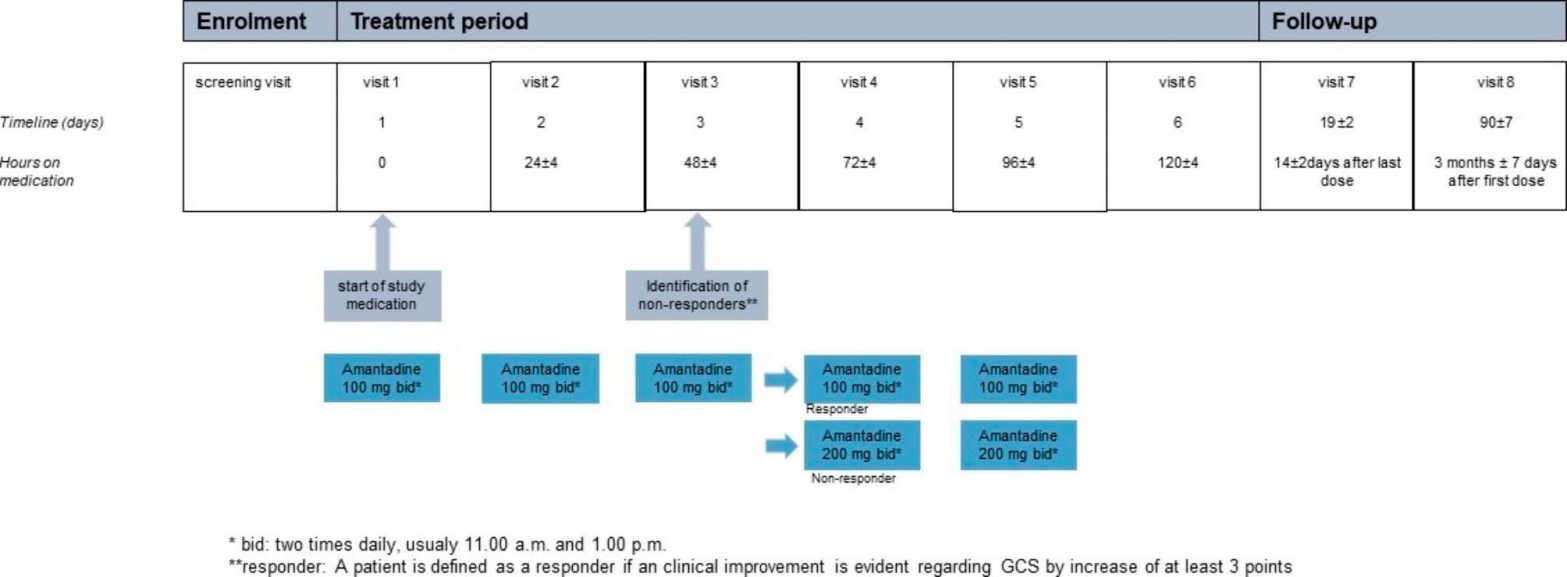
Overall study design

The following general procedures will be part of the study visits: patient history and demographics, documentation of clinical data (age, gender, handedness, intensive care unit and hospital length of stay, mortality, discharge disposition) and drug history, physical and neurological examination, blood tests (especially infection markers and electrolytes), electroencephalography (EEG), electrocardiography (ECG) as well as several questionnaires concerning consciousness and functional clinical outcome. For further details regarding the visit schedule, please see Fig. 1; Table 2. The majority of the concomitant measures are non-invasive and performed as part of the clinical routine in vigilance-reduced intensive care patients. Moreover, intensive and intermediate care unit patients are continuously monitored, so that possible adverse effects can be detected and treated immediately.

**Table 2:** Synopsis of planned study visits. The whole study duration for each subject will be 3 months incl. screening and follow-up procedures

	Enrolment		Treatment period					Follow-up	
	Screening/Consent	Visit 1 (baseline and first day of treatment)	Visit 2 (after 24 ± 4 h on amantadine)	Visit 3 (after 48 ± 4 h on amantadine)	Visit 4 (after 72 ± 4 h on amantadine)	Visit 5 (after 96 ± 4 h on amantadine)	Visit 6 (after 120 ± 4 h on amantadine)	Visit 7 (14 days after last dose)	Visit 8* (after 3 months ± 7 days from1st dose)
*Timeline (days)*		*1*	*2*	*3*	*4*	*5*	*6*	*-*	*90*
Informed consent	X								
Inclusion /exclusion criteria	X (using a screening sheet)	X (recheck)							
Patient history, demographics		X							
Documentation of clinical data		X							
Documentation of drug history		X							
Physical and Neurological examination		X							
Blood tests (approx. 10 ml)		X							
Start of trial drug		X							
Responsiveness check				X*					
Pregnancy Test (measurement of hCG in serum) in woman of child bearing potential	X								
GCS		X*	X	X*	X	X	X*	X	X
FOUR Score Coma Scale		X	X	X	X	X	X	X	X
RASS		X	X	X	X	X	X	X	X
ICDSC		X	X	X	X	X	X	X	X
EEG		X					X	X	
ECG		X	X	X	X	X	X	X	
mRS		X						X	X
NIHSS		X						X	X
GOS-E		X	X	X	X	X	X	X	X
CRS-R		X	X	X	X	X	X	X	X
MoCA		X							X
Questionnaire for therapists		X	X	X	X	X	X	X	(X)
Documentation of (S)AE		X	X	X	X	X	X	X	

Abbreviations: hCG: human Choriongonadotropin, GCS: Glasgow Coma Scale, FOUR: Full Outline UnResponsiveness, RASS: Richmond Agitation Sedation Scale, ICDSC: Intensive Care Delirium Screening Checklist, EEG: Electroencephalography, ECG: Electrocardiogram, mRS: modified Ranking Scale, NIHSS: National Institutes of Health Stroke Scale, GOS-E: Glasgow Outcome Scale-Extended, CRS-R: Coma Recovery Scale-Revised, MoCA: Montreal Cognitive Assessment, (S)AE: Serious Adverse Event

* Assessed by two independent clinical investigators who separately score the patient. In case of disagreement, a third independent investigator will perform the GCS and responsive check

### Who will take informed consent?

As the investigated study collective is per definition suffering from reduced consciousness, the patient’s legal representative will provide written informed consent. The informed consent of the patient will be sought retroactively as soon as possible. When the legal representative has not been named yet, a rapid application for a legal representative is initiated (in Germany called “Eilbetreuuung”).

### Additional consent provisions for collection and use of participant data and biological specimens

Not applicable.

### Interventions

#### Explanation for the choice of comparators

Not applicable.

### Intervention description

Intravenous amantadine at a dosage of 200 mg per day (100 mg each at 10:00 a.m. and 01:00 p.m.) will be applied for five days (120 h). If a patient presents as a non-responder (defined as an increase in GCS ≤ 2 points or a decrease in GCS) within the first 48 h after amantadine treatment initiation, the dosage will be increased after ECG control to 200 mg intravenously twice daily. Two independent clinical investigators score the patient separately to assess the primary endpoint. In case of disagreement, a third independent investigator will perform the GCS. Responding patients will remain on the 200 mg per day regime until day 5 (see Tables 2 and 3). This is in line with the recommendations for the dosage of amantadine in post-comatose patients with reduced vigilance (in traumatic brain lesions) (Summary of Product Characteristics (SmPC)) [42].

**Table 3:** Dose schedule

	Morning 10.00 am	Noon 1.00 p.m.	Evening	Night	note
Day 1	100 mg	100 mg	0	0	
Day 2	100 mg	100 mg	0	0	
Day 3	100–200 mg	100–200 mg	0	0	Dose can be doubled in case of unresponsiveness to drug; minimum duration of treatment
	200 mg	200 mg	0	0	In case of non-responder
Day 4	100 mg	100 mg	0	0	
	200 mg	200 mg	0	0	In case of non-responder
Day 5	100 mg	100 mg	0	0	Maximum duration of treatment
	200 mg	200 mg	0	0	In case of non-responder Maximum duration of treatment

The trial medication will be administered intravenously in a clinical setting. The site investigator will keep an account of the trial medication and acknowledge the receipt of all shipments of the trial medication. The investigator will document the date of the dispensary, subject identification, batch/ serial numbers or other identification of trial medication.

### Criteria for discontinuing or modifying allocated interventions 

#### Renal function impairment

As the renal clearance of amantadine is significantly lower in adult patients with moderate or severe renal impairment compared with healthy adults and the renal pathway is a major elimination pathway, impairment in renal function can result in significant accumulation in the plasma. In case of renal insufficiency, the dosage will be modified according to the dose adjustment table (see Table 4).

**Table 4:** Dose modification in case of renal insufficiency

Glomerular filtration rate (GFR) in ml/min	Dosage modification in mg	Administration interval
80 − 60	no dosage modification	
60 − 50	no dosage modification	
50 − 30	100 mg	1-1-0 No increase in non-responders
30 − 20	100 mg	1-0-0 no increase in non-responders
20 − 10	100 mg	1-0-0 no increase in non-responders
< 10 or hemodialysis	excluded from trial	

#### Allergic reactions

Amantadine might provide unknown allergic potential. If this is suspected withdraw the medication from the patient. This should be reported as an adverse event.

#### Toxicities

If treatment-related toxicities occur during the study, especially epileptic seizure, spasticity, ECG QTc > 480 ms, or any other suspicious ECG changes or cardial events (e.g., arrythmia, myocarditis, heart failure), drug administration will be terminated immediately. This should be reported as an adverse event.

If hallucinations occur, the dosage will be decreased to 50%. If successful, the administration will be continued with the reduced dosage, otherwise, it will be abandoned entirely. This should be reported as an adverse event.

#### Overdosage

The acute amantadine intoxication state is characterized by nausea, vomiting, hyperexcitability, tremor, ataxia, blurred vision, lethargy, depression, dysarthria and cerebral seizures; malignant cardiac arrhythmia has been reported in one case.

Acute toxic psychosis in the form of confusional/ delirium with visual hallucinations up to coma, as well as myoclonus, has been observed with concomitant administration of amantadine with other antiparkinsonian drugs.

No specific drug therapy or antidote is known in case of intoxication. In case of oral intake, vomiting should be induced or gastric lavage should be performed. In the case of vitally threatening intoxication, intensive monitoring measures are also required. Therapeutic options include fluid intake, acidification of the urine for faster excretion of the substance, sedation if necessary, anti-seizure medication, and antiarrhythmic drugs (lidocaine i.v.). To treat neurotoxic symptoms (as described above), intravenous administration of 1–2 mg physostigmine every 2 h can be tried in adults up to a maximum dose of 2 mg. Due to the low dialysability of amantadine (approx. 5%), hemodialysis is not advisable. It is recommended to monitor patients particularly with regard to a possible QT prolongation and factors favoring the occurrence of torsade de pointes, e.g., electrolyte disturbances (especially hypokalemia and hypomagnesaemia) or bradycardia.

### Strategies to improve adherence to interventions

Not applicable. ANNES Trial is performed in intensive care units, where research physicians control the medication every day.

### Relevant concomitant care permitted or prohibited during the trial

The administration of other medication prolonging QT time (e.g., Class IA and III antiarrhythmic drugs, antipsychotics, macrolide antibiotics) is not permitted during treatment with amantadine. If this is necessary due to a medical condition, QT time must be closely monitored. Moreover, administration of medication reducing consciousness (e.g., propofol, ketamine) should be avoid.

### Provisions for post-trial care

Patients that are enrolled in the ANNES study are insured through HDI Global SE in case of negligent harm or death during amantadine treatment. The maximum payment by the insurer for a single patient is limited to 500.000 Euro.

### Participant timeline

The individual study duration will be three months comprising of eight study visits in total (one screening visit, five visits during the treatment period and two follow-up visits, one two weeks after last medication intake and one at follow-up after three months).

### Statistical planning

#### Sample size calculation

The sample size is calculated using the two-stage optimum design of Simon for Phase II trials (see link below or nQuery Sample Size Software). Using this design, we proceed as follows: First we define a favorable true success rate of 40% successes which should lead with 80% power to a successful study. If this rate is 20% or smaller, the study should be with probability of at most 5% (type 1 error, one-sided) successful. We include 43 evaluable patients in the trial. After 13 patients, an interim analysis for futility is performed. The study is terminated early if less than 4 successes are observed. If four or more successes are observed, the study continues with 30 additional patients to recruit a total of 43 patients. According two-stage optimum design the final cut-off are 12 patients. Therefore, the study is successful if at least 13 of 43 patients show an increase of GCS score of at least 3 points (see supplemental Figs. 1 and 2):

#### Statistical analysis

The study is successful if a total of 13 of 43 patients show success (improvement of at least 3 points on the GCS). Using the two-stage optimum design of Simon for Phase II trials, non-evaluable patients have to be replaced. However, if the necessary number of 13 successes is achieved with less than 43 patients, there will be no early stopping for success. Calculations have been done with a Simon design calculator presented by the NIH on the internet: http://linus.nci.nih.gov/cgi-bin/simonr/cgi_main.

The primary endpoint *improvement of the GCS score from screening up to day 5 of at least 3 points* is analyzed according to the Simon design as described above. The primary analysis population is the per protocol population. This population includes all patients with GCS measurement at day 6 and all patients who died until day 6 (failures). No planned subgroup analyses will be performed. The secondary endpoints (mRS, NIHSS, GOS-E, CRS-R, MoCA after 90 days, RASS and ICDSC) will be analyzed by mixed models with time (categorially coded) as only factor including all measurements up to 3 months follow up. In case of severe violation of normal distribution assumption data will be analyzed by nonparametric tests (Friedmann with including pairwise comparisons using Nemenyis test). No imputation of missing values is planned. P-values will be computed, significances are only “local” in the sense that they are not corrected for multiple testing. The only confirmatory analysis is the one for the primary endpoint.

#### Interim analyses

After 13 patients, an interim analysis for futility is performed. The study is terminated early if less than 4 successes are observed. If four or more successes are observed, the study continues with 30 additional patients to recruit a total of 43 patients.

### Methods for additional analyses (e.g., subgroup analyses)

No planned subgroup analyses will be performed.

### Methods in analysis to handle protocol non-adherence and any statistical methods to handle missing data

No imputation of missing values is planned. The analysis population for the primary endpoint is defined as patients who show adherence to the study protocol to at least Visit 6 (GCS measurement after 5 days amantadine) and all patients who died until day 6 (failures). Dropouts before Visit 6 (e.g., anesthesia required) will be excluded from trial.

### Plans to give access to the full protocol, participant level-data and statistical code

The raw ANNES data is not available for public due to data privacy laws. However, the fully data collection is available from the authors upon reasonable request.

### Recruitment

Patients are recruited by research physicians on the intensive and intermediate care units within the department of neurology, internal medicine and anesthesiology of the University Hospital Tübingen by screening suitable subjects. The recruitment will be monitored in a screening and prescreening log. The duration of the recruitment period is approximately 16 months.

### Assignment of interventions: allocation

Not applicable.

### Assignment of interventions: blinding

Not applicable.

### Data collection and management

**Plans to promote participant retention and complete follow-up**.

Not applicable.

### Data management

All data will be collected in the CRF paper form at first. Authorized clinical staff at the investigational site will enter the data into the electronically CRF (eCRF) using an access controlled, audit-trailed, ICH/GCP compliant, validated system. Entered data will be subjected to plausibility, monitoring and medical review. Implausible or missing data will be queried. Database lock will be performed after completion of data entry, data cleaning and a final data review.

### Confidentiality

All human identifiable data will be stored in anonymized form using a sequential participant identifier. The correspondence between participant identifier and personal identifying information will be kept secure and accessible only where required.

### Data safety and monitoring board (DSMB)

The Data safety and monitoring board (DSMB) – also called data monitoring committee (DMC) is formed by the ZKS Tuebingen, Center for Clinical Studies. It is an independent group of experts in performing clinical trials who monitor patient safety and treatment efficacy data while our clinical trial is ongoing. If necessary, the DSMB can give recommendations to the principal investigators and sponsor of the trial for discontinuation, modification or continuation of the study. Reasons for individual study drug discontinuation or modification might be intolerance to study medication (please see section Trial intervention).

### Plans for collection, laboratory evaluation and storage of biological specimens for genetic or molecular analysis in this trail/future use

Not applicable.

## Oversight and monitoring

**Composition of the coordinating centre and trial streering committee**.

### Principal investigator

PD Dr. med. Annerose Mengel.

- designed the study protocol, revised the manuscript critically and is responsible for the electrophysiological analysis.
- organizing meetings of the study group.
- recruitment, data collection and completion of eCRFs.
- Adverse events reporting to ZKS Tuebingen, observance of AE-Reporting.
- monitoring data quality and conformity to protocol.

**Delegated of Sponsor and person in charge to meet the obligations of the sponsor**:

PD Dr. med. Katharina Feil.

- design and conduct of ANNES.
- designed the study protocol, revised the manuscript critically.
- organizing meetings of the study group.
- recruitment, data collection and completion of eCRFs.
- adverse events reporting to ZKS Tuebingen, observance of AE-Reporting.
- monitoring data quality and conformity to protocol.

### Study group/Research physicians

Dr. med. Kamaldeen Adeyemi, Dr. med. Corinna Blum, Dr. med. Tim W. Rattay, Constanze Single.

- revised the manuscript critically.
- recruitment, data collection and completion of eCRFs.
- adverse events reporting to ZKS Tuebingen.
- responsible for trial master file.

### Monitoring

Monika Ums, Dr. Manola Zago (ZKS Tuebingen, Center for Clinical Studies).

- monitoring data quality and conformity to protocol.
- observance of AE-Reporting.

### Datamanagement

Ruth Bösel, Dr. Bettina Brendel (IKEAB, Institute for Clinical Epidemiology and Applied Biometry).

### Biostatistician

Prof. Peter Martus.

**Composition of the data monitoring committee, its role and reporting structure**.

The data monitoring committee (DMC) is formed by the ZKS Tuebingen, Center for Clinical Studies. The DMC is independent from the principle investigator and sponsor. An interim-analysis is performed after 13 patients, undergoing amantadine treatment for at least 5 days. The study is terminated early if less than 4 successes are observed.

**Adverse event reporting and harms**.

**Safety assessment**.

Adverse events (AE), severe adverse events (SAE) and suspected unexpected serious adverse reactions (SUSAR) will be detected as exploratory endpoints within the study. A serious adverse event (SAE) means any untoward medical occurrence that at any dose:

- results in death.
- is life-threatening.
- requires inpatient hospitalization or prolongation of existing hospitalization.
- results in persistent or significant disability/ incapacity.
- results in a congenital anomaly / birth defect.
- is medically significant (e.g., suspected transmission of an infectious agent via medicinal product).

Planned hospitalizations or prolongation of existing hospitalization as a result of the following causes need not to be reported as SAE:

- drug application.
- test procedure required in the protocol.
- hospitalizations for diagnostic measures only.
- technical, practical, or social reasons, in absence of an adverse event.
- surgical intervention or other measures and the conditions leading to these measures are not AEs, if the condition leading to the measure was present prior to inclusion into the trial.
- stay at rehabilitation clinic.

Medical Dictionary of Regulatory Activities (MedDRA)-Coding will be used. All AE, SAE and SUSAR occurring after entry into the study and until day 14 after amantadine treatment (Visit 7) will be recorded. Within 24 h of occurrence, the adverse event has to be reported to the ZKS Tuebingen. The principle investigator or sponsor will determine relatedness of an event to study drug and decides potential termination of Amantadine treatment. The study will monitor for the following adverse events daily during patient examination: epileptic seizure, spasticity, ECG QTc > 480 ms, arrythmia, heart failure and hallucinations.

### Frequency and plans for auditing trial conduct

No auditing is planned.

### Plans for communicating important protocol amendments to relevant parties (e.g., trial participants, ethical committees)

Any modifications of the procotol including changes to primary or secondary outcome, sample size calculation, eligibility criteria, study procedures and statistical analysis will require an amendment to the protocol. This amendment will be approved by the local ethics committee.

### Dissemination plans

Study results will be reported by publication in a pubmed listed medical journal. We would like to emphasize, that the results will be reported regardless of the magnitude or direction of effect. Individual patients will be informed of the results of the completed study upon request.

## Discussion

Disorders of consciousness with persisting coma are presenting a major challenge in the clinical practice of neurointensive care patients. Despite the lack of high-quality data supporting efficacy of pharmacological intervention targets in neurotransmitter pathways, medication for neuroenhancement is commonly used to support rehabilitation of patients with disorders of consciousness [44]. To present, no treatment recommendation can be made regarding persisting coma in neurointensive care patients as clinical trials are generally lacking. Moreover, pharmacological treatments proposed and discussed for disorders of consciousness target heterogenous aspects, i.e., awareness, consciousness, responsivity, survival and functional outcome. In the area of traumatic brain injury, there are prospective data available that have led to treatment recommendations [45]. However, only one trial on amantadine in traumatic brain injury was methodologically robust, although affected by limitations. The main outcome measured neurological recovery rather than consciousness, but amantadine lost efficacy at post-treatment follow-up, and narcotics were used more in the placebo group [21].

Within the field of non-traumatic brain injuries, especially strokes, there only exists good clinical experience as well as promising retrospective and review [22, 29, 46] data. Hence, there is a significant unmet need for controlled and prospective datasets within clearly defined patient collectives. We assume that within such a study the amantadine effect on the enhancement of vigilance in non-traumatic brain injury can be objectified. The advantage of an increased vigilance is improved participation in early rehabilitation, leading to a better functional outcome for the patients. This relevant potential benefit of the study clearly outweighs the putative risk of amantadine, a medication that has been known for many years, for instance from the treatment of Parkinson’s Disease. Further, the well-known side effects described above can be anticipated and well handled. Beyond this assumed individual benefit it is expected that the results of this study will be of significant long-term importance to improve therapeutic standards in acute neurointensive care unit patients in general. Indeed, recently, one of the first comprehensive studies in this field [46] has shown that amantadine medication leads to an improvement of vigilance even in patients with non-traumatic brain injuries (especially those with AIS, ICH and SAH) after five days of treatment. The authors concluded that these results should be confirmed in further studies also considering the functional outcome of patients which will be part of our secondary endpoints. Further, they emphasized that epileptic seizures should be considered as a main side effect of amantadine treatment, which we will try to strictly control for via repeated EEG analyses. On the other hand, the risk of increased seizure activity and decreased convulsive threshold emerged after reports of seizures induced in the setting of drug overuse. More recent reports in adult and pediatric patients with either traumatic brain injury or epilepsy and amantadine therapy suggest that there is either no difference or even a decreased seizure incidence in patients receiving stimulant therapy [21, 28, 47]. Overall, in the clinical setting, the potential benefit in enhancing functional recovery and consciousness outweighs the risk of seizure exacerbation. Additionally, our patients will be monitored closely during the initiation of therapy. The longitudinal EEG recordings will be used not only to monitor adverse events, but also changes in consciousness.

From a methodological point of view, one shortcoming of the current study might be that the GCS is very well established and easy to conduct on the one hand but may lead to practical limitation in intubated patients on the other hand. This is why we plan to perform several other neuropsychological test scores, too, with the goal to validate the optimal endpoint within this treatment design. Further, it will be important for the success of the *ANNES* study that reduced vigilance must not be explained to a significant extent via a known mechanism, e.g., sepsis, disturbance of electrolytes or status epilepticus, which are common in intensive care unit patients, as well. However, in nearly all of the above-mentioned situations it will be crucial to untangle the effects of a reduced level of consciousness directly induced by the primary disease from those associated with a superimposed delirium [48]. This is why, we plan to regularly perform laboratory analyses, EEG analysis and the ICDSC score. Furthermore, for assessment of responsiveness to medical neuroenhancement it finally might be crucial, where appropriate, to stratify the patients according to the anatomical location of their CNS lesion [49].

Ethically, a characteristic challenge of the *ANNES* will be the receipt of informed consent as the investigated study collective is per definition suffering from reduced consciousness and therefore not in a position to provide written informed consent. It is planned that the patient’s legal representative will give written informed consent. Inclusion of a patient is also possible if two independent physicians agree to include the patient in the study. In this case the informed consent of the patient or his/her legal representative will be sought retroactively as soon as possible. In case of regained consciousness, the subject has the right to object to study participation at any time. The subject’s participation in the study is then terminated immediately.

In summary, the goal of pharmacologic neuroenhancement therapy using amantadine in neurointensive care patients with acquired, non-traumatic lesions is centered around the restoration of the balance of neurotransmitter networks to accelerate recovery of neurological function, stimulate emergence from disorders of consciousness and to maximize functional outcome. The importance of supporting patient recovery and rehabilitation cannot be overstated as early and intensive neurorehabilitation can improve long-term functional outcome [50]. ANNES as an investigator-initiated, monocenter, phase IIb, proof of concept, open-label pilot study will provide important information and data in the context of disorders of consciousness in neurointensive care patients with non-traumatic brain injuries. These data can be used for a following placebo-controlled study to investigate the efficacy of amantadine in neurointensive care patients with persisting coma and disorders of consciousness regarding homogenous patient population, and the choice of the right outcome parameter toward motor, cognitive or functional domains. The GCS as commonly used outcome measure could be insensitive to detect subtle changes in neurobehavioral function, therefore we will examine different outcome scales to identify accurately whether a patient is responsive to neuroenhancement therapy. Finally, changes in the state of consciousness will be objectified by repeated EEG measurements providing valuable data for the application of resting-state EEG for the diagnosis of disorders of consciousness and outcome prediction. The data collected in this process may yield important insights in the pathophysiology of impaired consciousness giving implications for new therapy options.

### Trial status

The current Protocol Version is No. 3.0, 2023-Feb-22, Amendment No.4. Recruitment began on 21st March 2023. The duration of the recruitment period is approximately 16 months.

### Electronic supplementary material

Below is the link to the electronic supplementary material.

Supplementary Material 1

## Data Availability

The datasets used and analysed during the current study are available from the corresponding author on reasonable request.

## References

[1] Kondziella D, Bender A, Diserens K, van Erp W, Estraneo A, Formisano R (2020). European Academy of Neurology guideline on the diagnosis of coma and other disorders of consciousness. Eur J Neurol.

[2] Laureys S, Celesia GG, Cohadon F, Lavrijsen J, León-Carrión J, Sannita WG (2010). Unresponsive wakefulness syndrome: a new name for the vegetative state or apallic syndrome. BMC Med.

[3] Giacino JT, Ashwal S, Childs N, Cranford R, Jennett B, Katz DI (2002). The minimally conscious state: definition and diagnostic criteria. Neurology.

[4] Kondziella D, Frontera JA, Pearls (2021). Oy-sters: eyes-open coma. Neurology.

[5] Li J, Zhang P, Wu S, Yuan R, Liu J, Tao W (2020). Impaired consciousness at stroke onset in large hemisphere infarction: incidence, risk factors and outcome. Sci Rep.

[6] Jang SH, Chang CH, Jung YJ, Kim JH, Kwon YH (2019). Relationship between impaired consciousness and Injury of Ascending Reticular activating system in patients with Intracerebral Hemorrhage. Stroke.

[7] Jang SH, Kim HS (2015). Aneurysmal Subarachnoid Hemorrhage causes Injury of the Ascending Reticular Activating System: relation to consciousness. AJNR Am J Neuroradiol.

[8] Suarez JI, Tarr RW, Selman WR (2006). Aneurysmal Subarachnoid Hemorrhage. N Engl J Med.

[9] Kumar S, Selim MH, Caplan LR (2010). Medical complications after stroke. Lancet Neurol.

[10] Holloway RG, Arnold RM, Creutzfeldt CJ, Lewis EF, Lutz BJ, McCann RM (2014). Palliative and end-of-life care in stroke: a statement for healthcare professionals from the American Heart Association/American Stroke Association. Stroke.

[11] Lutkenhoff ES, Chiang J, Tshibanda L, Kamau E, Kirsch M, Pickard JD (2015). Thalamic and extrathalamic mechanisms of consciousness after severe brain injury: Brain Injury and consciousness. Ann Neurol.

[12] Young GB (2009). Coma. Annals of the New York. Acad Sci.

[13] Clarençon F, Bardinet É, Martinerie J, Pelbarg V, Menjot de Champfleur N, Gupta R et al. Lesions in deep gray nuclei after severe traumatic brain injury predict neurologic outcome. Mohapatra S, editor. PLoS ONE. 2017;12(11):e0186641. 10.1371/journal.pone.0186641 PMC5667824 29095850

[14] Brown EN, Lydic R, Schiff ND. General Anesthesia, Sleep, and Coma. Schwartz RS, editor. N Engl J Med. 2010;363(27):2638–50. 10.1056/NEJMra0808281 PMC3162622 21190458

[15] Edlow BL, Sanz LRD, Polizzotto L, Pouratian N, Rolston JD, Snider SB (2021). Therapies to restore consciousness in patients with severe brain injuries: a gap analysis and future directions. Neurocrit Care.

[16] Danysz W, Dekundy A, Scheschonka A, Riederer P (2021). Amantadine: reappraisal of the timeless diamond—target updates and novel therapeutic potentials. J Neural Transm.

[17] Farnebo LO, Fuxe K, Goldstein M, Hamberger B, Ungerstedt U (1971). Dopamine and noradrenaline releasing action of amantadine in the central and peripheral nervous system: a possible mode of action in Parkinson’s disease. Eur J Pharmacol.

[18] Volonté MA, Moresco RM, Gobbo C, Messa C, Carpinelli A, Rizzo G (2001). A PET study with [11-C]raclopride in Parkinson’s disease: preliminary results on the effect of amantadine on the dopaminergic system. Neurol Sci.

[19] Mizoguchi K, Yokoo H, Yoshida M, Tanaka T, Tanaka M (1994). Amantadine increases the extracellular dopamine levels in the striatum by re-uptake inhibition and by N-methyl-d-aspartate antagonism. Brain Res.

[20] Giacino JT, Katz DI, Schiff ND, Whyte J, Ashman EJ, Ashwal S (2018). Practice guideline update recommendations summary: Disorders of consciousness: report of the Guideline Development, Dissemination, and implementation Subcommittee of the American Academy of Neurology; the American Congress of Rehabilitation Medicine; and the National Institute on Disability, Independent Living, and Rehabilitation Research. Neurology.

[21] Giacino JT, Whyte J, Bagiella E, Kalmar K, Childs N, Khademi A (2012). Placebo-controlled trial of amantadine for severe traumatic brain Injury. N Engl J Med.

[22] Gagnon DJ, Leclerc AM, Riker RR, Brown CS, May T, Nocella K (2020). Amantadine and Modafinil as Neurostimulants during Post-stroke Care: a systematic review. Neurocrit Care.

[23] Herrold AA, Pape TLB, Guernon A, Mallinson T, Collins E, Jordan N (2014). Prescribing multiple neurostimulants during Rehabilitation for severe Brain Injury. Sci World J.

[24] Jibiki I, Morikawa K, Yamaguchi N (1993). Beneficial effect of amantadine on symptoms of dementia in patients with cerebral infarctions. Acta Ther.

[25] Landucci E, Filippi L, Gerace E, Catarzi S, Guerrini R, Pellegrini-Giampietro DE (2018). Neuroprotective effects of topiramate and memantine in combination with hypothermia in hypoxic-ischemic brain injury in vitro and in vivo. Neurosci Lett.

[26] Gower A, Tiberi M (2018). The intersection of central dopamine system and stroke: potential avenues aiming at Enhancement of Motor Recovery. Front Synaptic Neurosci.

[27] Winstein CJ, Stein J, Arena R, Bates B, Cherney LR, Cramer SC (2016). Guidelines for adult Stroke Rehabilitation and Recovery: a Guideline for Healthcare Professionals from the American Heart Association/American Stroke Association. Stroke.

[28] Ghalaenovi H, Fattahi A, Koohpayehzadeh J, Khodadost M, Fatahi N, Taheri M (2018). The effects of amantadine on traumatic brain injury outcome: a double-blind, randomized, controlled, clinical trial. Brain Injury.

[29] Leclerc AM, Riker RR, Brown CS, May T, Nocella K, Cote J (2021). Amantadine and Modafinil as Neurostimulants following Acute Stroke: a retrospective study of Intensive Care Unit Patients. Neurocrit Care.

[30] Wijdicks EFM, Bamlet WR, Maramattom BV, Manno EM, McClelland RL (2005). Validation of a new coma scale: the FOUR score. Ann Neurol.

[31] Sessler CN, Gosnell MS, Grap MJ, Brophy GM, O’Neal PV, Keane KA (2002). The Richmond agitation-sedation scale: validity and reliability in adult intensive care unit patients. Am J Respir Crit Care Med.

[32] Jennett B, Snoek J, Bond MR, Brooks N (1981). Disability after severe head injury: observations on the use of the Glasgow Outcome Scale. J Neurol Neurosurg Psychiatry.

[33] Wilson L, Boase K, Nelson LD, Temkin NR, Giacino JT, Markowitz AJ (2021). A manual for the Glasgow Outcome Scale-Extended interview. J Neurotrauma.

[34] Giacino JT, Kalmar K, Whyte J, The, JFK Coma Recovery Scale-Revised (2004). Measurement characteristics and diagnostic utility11No commercial party having a direct financial interest in the results of the research supporting this article has or will confer a benefit upon the authors or upon any organization with which the authors are associated. Arch Phys Med Rehabil.

[35] Bergeron N, Dubois MJ, Dumont M, Dial S, Skrobik Y (2001). Intensive care Delirium Screening Checklist: evaluation of a new screening tool. Intensive Care Med.

[36] Wilson JTL, Hareendran A, Grant M, Baird T, Schulz UGR, Muir KW (2002). Improving the assessment of outcomes in stroke: use of a structured interview to assign grades on the modified Rankin Scale. Stroke.

[37] Kwah LK, Diong J (2014). National Institutes of Health Stroke Scale (NIHSS). J Physiother.

[38] Nasreddine ZS, Phillips NA, BÃ©dirian V, Charbonneau S, Whitehead V, Collin I (2005). The Montreal Cognitive Assessment, MoCA: a brief Screening Tool for mild cognitive impairment: MOCA: a BRIEF SCREENING TOOL FOR MCI. J Am Geriatr Soc.

[39] Hankemeier A, Rollnik JD (2015). The early functional abilities (EFA) scale to assess neurological and neurosurgical early rehabilitation patients. BMC Neurol.

[40] Duszyk-Bogorodzka A, Zieleniewska M, Jankowiak-Siuda K (2022). Brain activity characteristics of patients with Disorders of consciousness in the EEG resting state paradigm: a review. Front Syst Neurosci.

[41] Bai Y, Lin Y, Ziemann U (2021). Managing disorders of consciousness: the role of electroencephalography. J Neurol.

[42] Fachinforation. Amantadin-ratiopharm 200 mg Infusionslösung. 2018.

[43] Leonardi M, Sattin D, Raggi A (2013). An italian population study on 600 persons in vegetative state and minimally conscious state. Brain Injury.

[44] Barra ME, Edlow BL, Brophy GM (2022). Pharmacologic therapies to promote recovery of consciousness. Semin Neurol.

[45] Loggini A, Tangonan R, El Ammar F, Mansour A, Goldenberg FD, Kramer CL (2020). The role of amantadine in cognitive recovery early after traumatic brain injury: a systematic review. Clin Neurol Neurosurg.

[46] Rühl L, Kuramatsu JB, Sembill JA, Kallmünzer B, Madzar D, Gerner ST (2022). Amantadine treatment is associated with improved consciousness in patients with non-traumatic brain injury. J Neurol Neurosurg Psychiatry.

[47] Meythaler JM, Brunner RC, Johnson A, Novack TA (2002). Amantadine to Improve Neurorecovery in Traumatic Brain Injury–Associated diffuse Axonal Injury: a pilot double-blind Randomized Trial. J Head Trauma Rehabilitation.

[48] Reznik ME, Yaghi S, Jayaraman MV, McTaggart RA, Hemendinger M, Mac Grory BC (2018). Level of consciousness at discharge and associations with outcome after ischemic stroke. J Neurol Sci.

[49] Brioschi A, Gramigna S, Werth E, Staub F, Ruffieux C, Bassetti C (2009). Effect of Modafinil on subjective fatigue in multiple sclerosis and stroke patients. Eur Neurol.

[50] Askim T, Bernhardt J, Salvesen Ø, Indredavik B (2014). Physical activity early after Stroke and its association to functional outcome 3 months later. J Stroke Cerebrovasc Dis.

