# Supplementary material for: Amantadine for NeuroenhaNcement in acutE patients Study - a protocol for a prospective pilot proof of concept phase IIb study in intensive and intermediate care unit patients (ANNES)
Source: BMC Neurol. 2023 Aug 22;23:308. doi: 10.1186/s12883-023-03345-w (PMC10464325; doi:10.1186/s12883-023-03345-w)
Supplement: Supplementary file 1 — Supplementary Material 1 [file 12883_2023_3345_MOESM1_ESM.docx]

**Supplementary Material**

Supplemental figure 1: Sample Size Calculation using two-stage optimum design of Simon for Phase II trials (performed by nQuery Sample Size Software)


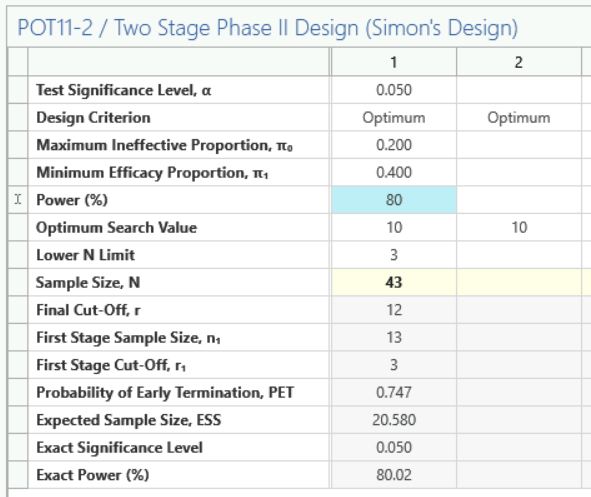


Supplemental figure 2: The Simon two-stage optimum design algorithm of the study
